# Procollagen type I N-terminal propeptide (PINP) is a marker for fibrogenesis in bile duct ligation-induced fibrosis in rats

**DOI:** 10.1186/1755-1536-3-5

**Published:** 2010-04-01

**Authors:** Sanne Skovgård Veidal, Efstathios Vassiliadis, Anne-Christine Bay-Jensen, Gervais Tougas, Ben Vainer, Morten Asser Karsdal

**Affiliations:** 1 Department of Pharmacology, Nordic Bioscience, Herlev, Denmark; 2 Department of Pathology, Rigshospitalet, University of Copenhagen, Copenhagen, Denmark; 3 Novartis Pharma AG Translational Medicine, Basel, Switzerland

## Abstract

**Background:**

Fibrosis can be described as the excess deposition of extracellular matrix (ECM) components, such as collagens and proteoglycans. Fibrosis of the liver, which eventually leads to cirrhosis, is a major global health problem. Being able to measure fibrosis progression may enable timely preventative intervention. The aim of the current study was to investigate the utility of serum procollagen type I N-terminal propeptide (PINP) as a marker of hepatic fibrosis, as distinct from bone formation, during three different periods of fibrosis development following hepatic injury induced by bile duct ligation (BDL) in rats.

**Methods:**

BDL was performed on 30 female Sprague-Dawley rats aged 6 months, and sham operations on 30 controls. Animals were killed after 14, 28, or 35 days. The extent of liver fibrosis was evaluated by quantitative histology after Sirus Red staining. Levels of serum PINP and osteocalcin (a marker solely for osteoblastic bone formation) were determined using ELISA at baseline and post termination.

**Results:**

Collagen formation increased by 30% compared to 3% in sham-operated animals (*P *< 0.0001). PINP levels increased significantly in all BDL groups compared with baseline (14 days: baseline 13.9 ng/ml, termination 17.7 ng/ml, *P *= 0.047; 28 days: baseline 17.9 ng/ml, termination 26.2 ng/ml, *P *= 0.005; 35 days: baseline 18.0 ng/ml, termination 27.4 ng/ml *P *= 0.015, an increase of 52%). PINP levels did not change from baseline in the sham-operated rats, indicating that the increased PINP levels were due to hepatic injury. The bone-specific marker, osteocalcin, did not increase in either BDL or sham-operated rats. PINP measured in serum correlated to the extent of liver fibrosis as evaluated by quantitative histology (R^2 ^= 0.42, *P *< 0.001).

**Conclusion:**

PINP was associated with the development of liver fibrosis, but not bone formation, in mature rats subjected to BDL. Thus, PINP may be useful in studying the pathogenesis of liver fibrosis. However, caution should be applied when interpreting PINP levels in other disease states.

## Background

Fibrosis may be described as extensive scar formation, observed as increased deposition and abnormal distribution of extracellular matrix (ECM) components, such as collagen and proteoglycans. Liver fibrosis is a serious complication of chronic inflammatory liver diseases arising from diverse infectious, inflammatory or toxic causes [1]. Progression of fibrosis eventually leads to liver cirrhosis, which is a major global health problem accounting for approximately 800,000 deaths per year worldwide [1-3].

Histopathological examination of liver biopsies is the gold standard for diagnosis and staging of chronic liver diseases and is of significance when evaluating the effect of therapeutic intervention. Liver biopsy, however, has two significant drawbacks. It is invasive, and prone to variation in the length and size of the tissue specimen which leads to low reproducibility and high intrapatient variation [4,5]. Therefore, the development of non-invasive biomarkers of fibrogenesis may be important for the staging and monitoring of chronic liver disease, and may provide additional information on the pathogenesis.

Matrix remodelling is a normal integrated process of tissue homeostasis and maintenance but it changes under certain disease conditions [6,7]. The central pathological feature of fibrosis is uncontrolled ECM remodelling [4-6,8,9]. The liver ECM is composed mainly of fibrous proteins such as collagens and proteoglycans [6,7,10]. During fibrogenesis the quality, quantity, and distribution of the ECM in the liver changes, which results in excessive accumulation of fibrous tissue (that is, scar tissue), and an overall increase in ECM density [6,7,10]. A cirrhotic liver may contain up to six times more collagen than that of a healthy liver [6-8,11].

The formation and degradation of ECM components is accompanied by the release of protein breakdown products into the circulation [7,12]. Thus, circulating levels of these byproducts may potentially be used as biochemical markers for assessing the extent of disease and prognosis, and for monitoring response to treatment [13]. Although several biomarkers such as hyalronic acid, collagen markers, and laminin are available for diagnosis and follow-up of liver fibrosis, the accuracy of these biomarkers for detection of fibrosis is highly variable [4,5].

In the healthy human liver the most abundant collagens are the fibril-forming types I and III. Fibril-forming collagens are synthesised as precursor molecules with large propeptide extensions at both the N-terminal and C-terminal of the molecule [14]. The mature propeptides are cleaved from procollagen by N-terminal or C-terminal proteinases, and mature collagen is integrated into the ECM [8,15,16]. During fibrogenesis, type I collagen levels increase up to eightfold [4,5,9,17]. Notably, type I collagen levels increase significantly more than type III, changing the ratio from 1:1 in the healthy liver to 1:2 in the cirrhotic liver [4,5,9,17]. Measuring serum levels of the procollagen type I N-terminal propeptide (PINP) released during collagen formation may be useful as a marker of fibrogenesis, either alone, in ratios or in combination with other techniques.

Several animal models for liver fibrosis have been developed, most of them using small rodents [11], each with individual strengths and weaknesses. Bile duct ligation (BDL) has been used as an animal model of chronic liver injury due to its resemblance to hepatocyte damage, hepatic stellate cell activation, and liver fibrosis observed in human cholestatic liver disease [18]. Even though a range of investigators have used BDL rats as models of liver fibrosis, the measurement of serological biochemical markers of liver ECM turnover has not been presented. This may in part be due to the lack of procollagen markers for rodent use. The current study is, to our knowledge, the first to use the type I collagen turnover marker, PINP, to monitor the development of liver fibrosis in BDL rats. In contrast to previous researchers, we used mature rats of 6 months of age, because in young rodents collagen turnover is highly elevated during skeletal growth and remodelling of the growth plate [12].

Bone turnover can be measured by serological biochemical markers [13]. Bone formation can be assessed by both type I collagen propeptides and osteocalcin, which is synthesised and secreted by osteoblasts during bone formation [12]. Collagen type I constitutes 90% of bone, but it is also present in many other tissues including liver, skin and tendons [19-21]. In contrast, osteocalcin is one of the major non-collagenous proteins of bone constituting approximately 50% of the total non-collagenous proteins [12], and is considered more bone specific.

The aim of the current study was to investigate whether PINP levels, indicating collagen type I formation, could be a potential marker for liver fibrosis in an experimental model. We compared levels of PINP with those of osteocalcin, a bone-specific marker, to investigate the specificity for liver and bone. We evaluated liver fibrosis by quantitative histology.

## Methods

### Animals

A total of 60 female Sprague-Dawley rats aged 6 months were housed at the animal research facilities at Nordic Bioscience, Denmark. The experiments were approved by the Experimental Animal Committee of the Danish Ministry of Justice, and were performed according to the European Standard for Good Clinical Practice (2008/561-1450). The rats were housed in standard type III-H cages at 18°C to 22°C with bedding and nest material (Altromin 1324; Altromin, Lage, Germany) and purified water (Milli-Q system; Millipore, Glostrup, Denmark) *ad libitum*. Rats were kept under conditions of a 12-h light: dark cycle. Experiments began after 1 week of acclimatisation.

Liver fibrosis was induced in anaesthetised rats by standard BDL in which the bile duct was ligated in two places and dissected between the ligations prior to closing the abdomen. In sham-operated rats, the abdomen was closed without BDL.

### Study design

The rats were divided into three groups: group 1 (10 BDL and 10 sham-operated rats) were killed after 2 weeks, group 2 (10 BDL and 10 sham-operated rats) were killed after 4 weeks, and group 3 (10 BDL and 10 sham-operated rats) were killed after 5 weeks. The procedure began with fasting the animals for at least 14 h, after which they were asphyxiated by CO_2 _and killed by exsanguination.

### Blood sampling

Blood samples were taken under light CO_2_/O_2 _anaesthesia at baseline and at termination from the retro-orbital sinus of rats that had fasted for at least 14 h. The collected blood was left for 30 min at room temperature to clot, followed by centrifugation at 1500 *g *for 10 min. All clot-free liquid was transferred to new tubes and centrifuged again at 1500 *g *for 10 min. The serum was then transferred to clean tubes and stored at -80°C until analysis was performed.

### Tissue handling

After the rats were killed, their livers were carefully dissected, weighed, fixed in 4% formaldehyde for a minimum of 24 h, cut into appropriate slices and embedded in paraffin. Sections 5 μm thick were cut, mounted on glass slides and stained with Masson's trichrome. The liver sections were evaluated histologically by assessment of the architecture, presence of inflammation, proliferation of bile ducts and fibrosis. The *de novo *bile duct formation in the parenchyma was evaluated semiquantitatively using the following scoring system: normal = 0, mild changes (one-third or less of the lobule affected) = 1, moderate changes (between one-third and two-thirds of the lobule affected) = 2, and severe changes (two-thirds or more of the lobule affected) = 3. Digital photographs were taken using an Olympus B × 60 microscope with × 40 and × 100 magnification and an Olympus 5050-zoom digital camera (Olympus, Tokyo, Japan).

### Histology image analysis

Histology sections stained with Sirus Red were analysed using Visiopharm software V. 3.2.8.0 (Hørsholm, Denmark). Images were acquired using a PixeLINK PL-A623C microscope digital camera (PixeLINK, Ottawa, Canada).

### Immunohistochemistry

Liver sections (5 μm) were deparaffinised, hydrated and further peroxidase activity was blocked with the addition of 0.4% hydrogen peroxide. Sections were then incubated with a polyclonal antibody against type I collagen (1:10; Abcam, Cambridge, UK). Sections were then rinsed and the antibody binding was depicted using the Super Sensitive Polymer-HRP IHC Detection System combined with AEC substrate, according to the supplier's instructions (Biogenex, Taby, Sweden). Sections were counterstained with Mayer's haematoxylin. Pictures were taken at × 33 magnification as described above.

### Determination of serum PINP and osteocalcin

The concentration of PINP was assayed using a commercial Rat PINP ELISA Kit (IDS Nordic, Herlev, Denmark), and osteocalcin was assayed using the commercial Osteocalcin ELISA kit (IDS Nordic). All samples were assayed in duplicate.

### Statistical analysis

Mean values and standard error of the mean (SEM) were calculated using GraphPad Prism (GraphPad Software, San Diego, CA, USA) and statistical significance assessed using the Student two-tailed paired t test (α = 0.05), assuming normal distribution, or the Mann-Whitney two-tailed non-parametric test (α = 0.05). The coefficient of correlation (R^2^) and the corresponding *P *value were determined by linear regression.

## Results

### Animals

During the 5 weeks, 6 of 60 rats, all of them BDL operated, were killed due to excessive weight loss.

### Gross liver appearance and histopathological findings

**Figure 1:**
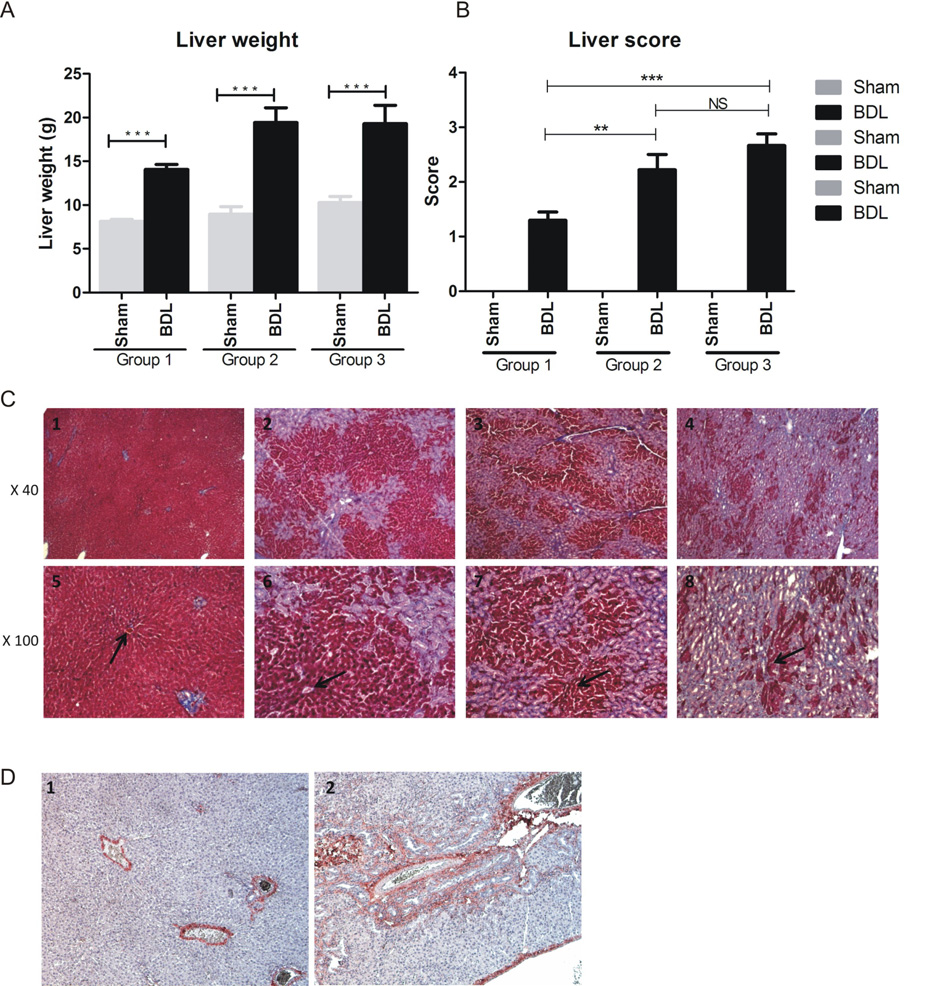
**Rat liver results**. **(a) **Liver weight in bile duct ligated (BDL) or sham-operated rats. Data are shown as mean ± standard error of the mean (SEM). ***P < 0.0001. **(b) **Scoring of the structural changes in the livers of each group. Data are shown as mean ± SEM. **(c) **Masson's trichrome photomicrographs showing the hepatic structure in rats 5 weeks after a sham operation (c2), 2 weeks after BDL (c3), 4 weeks after BDL (c4) and 5 weeks after BDL (c5). The structure of the hepatic lobules around the portal tract (arrows) is clearly disrupted in the BDL rats compared with the sham-operated rats. **(d) **Immunohistochemical analysis of type I collagen. Type I collagen is localised around fibrotic structures. Original magnification × 40 or × 100.

At the time of death, the livers of control animals showed normal gross morphology while the livers of BDL animals were enlarged. Mean liver weights were significantly increased in BDL rats compared to the sham-operated controls (group 1: sham 8.1 g, BDL 14.1 g; group 2: sham 9.0 g, BDL 19.4 g; group 3: sham 10.3 g, BDL 19.3 g) (Figure 1a). Semiquantitative scoring of liver sections using the 0-3 scale showed significantly more structural changes of the livers in groups 2 and 3 compared to group 1 (Figure 1b). Histological examination of the livers of sham-operated animals showed they were microscopically normal, with no sign of fibrosis (Figure 1c, panels 1-4). In BDL livers, a marked ductal proliferation was observed. In group 1 (Figure 1c, panels 5-8) the proliferation was located around the portal tract while in group 3 (Figure 1c, panel 8) the proliferation had spread. Collagen deposition was found around the ductular structures (Figure 1c, panels 5-8). Inflammation was minimal and confined to the portal tracts. No other signs of cholestasis were seen, whether intracellular cholestasis, bile plugs, bile infarction or hepatocytic rosette formation.

By immunohistochemistry, collagen type I deposition was found exclusively in the venous wall of healthy rats (Figure 1d). In contrast, in group 3 rats in which marked ductal proliferation was seen around the portal tract with the formation of multiple neo-bile ducts, more extensive type I collagen was found (Figure 1d, panel 1).

### Serological markers of PINP and osteocalcin

**Figure 2:**
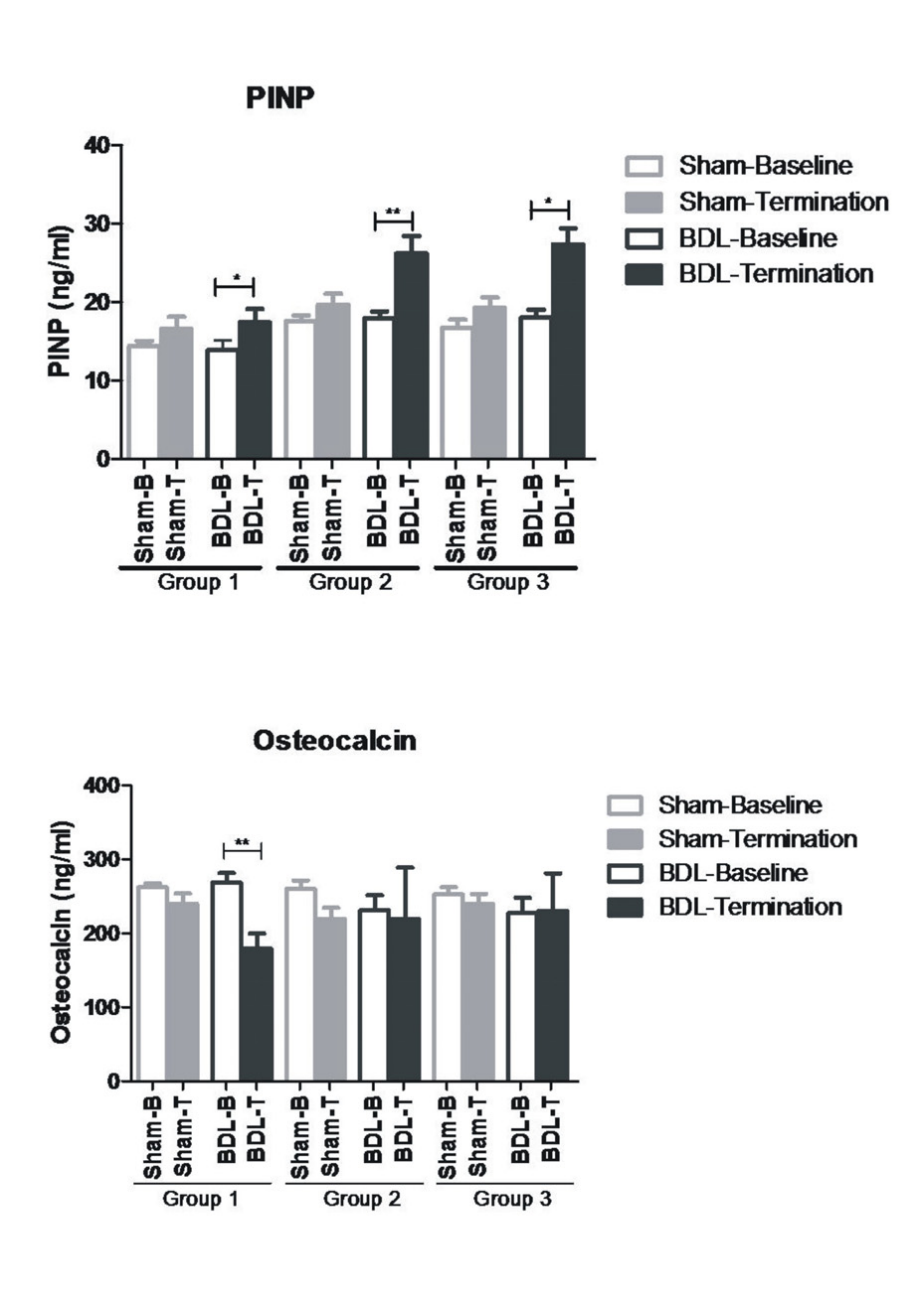
**Procollagen and osteocalcin levels**. **(a) **Serum procollagen type I N-terminal propeptide (PINP) levels in bile duct ligated (BDL) or sham-operated rats. Data are shown as mean ± standard error of the mean (SEM). **(b) **Serum osteocalcin levels in bile duct ligated (BDL) or sham-operated rats. Data are shown as mean ± SEM.

PINP levels increased significantly in all BDL groups compared with baseline (group 1: baseline 13.9 ng/ml, termination 17.4 ng/ml; group 2: baseline 17.9 ng/ml, termination 26.2 ng/ml; group 3: baseline 18.0 ng/ml, termination 27.4 ng/ml) (14 days: *P *= 0.047; 24 days: *P *= 0.005; 35 days: *P *= 0.015; with the maximum increase of 52% seen in group 3) (Figure 2a). PINP levels did not change significantly in the sham-operated rats (Figure 2a).

In contrast to PINP, the bone-specific marker osteocalcin did not increase from baseline in BDL or in sham-operated rats during the study period, nor did it differ over time following BDL (Figure 2b).

### Total collagen increase

**Figure 3:**
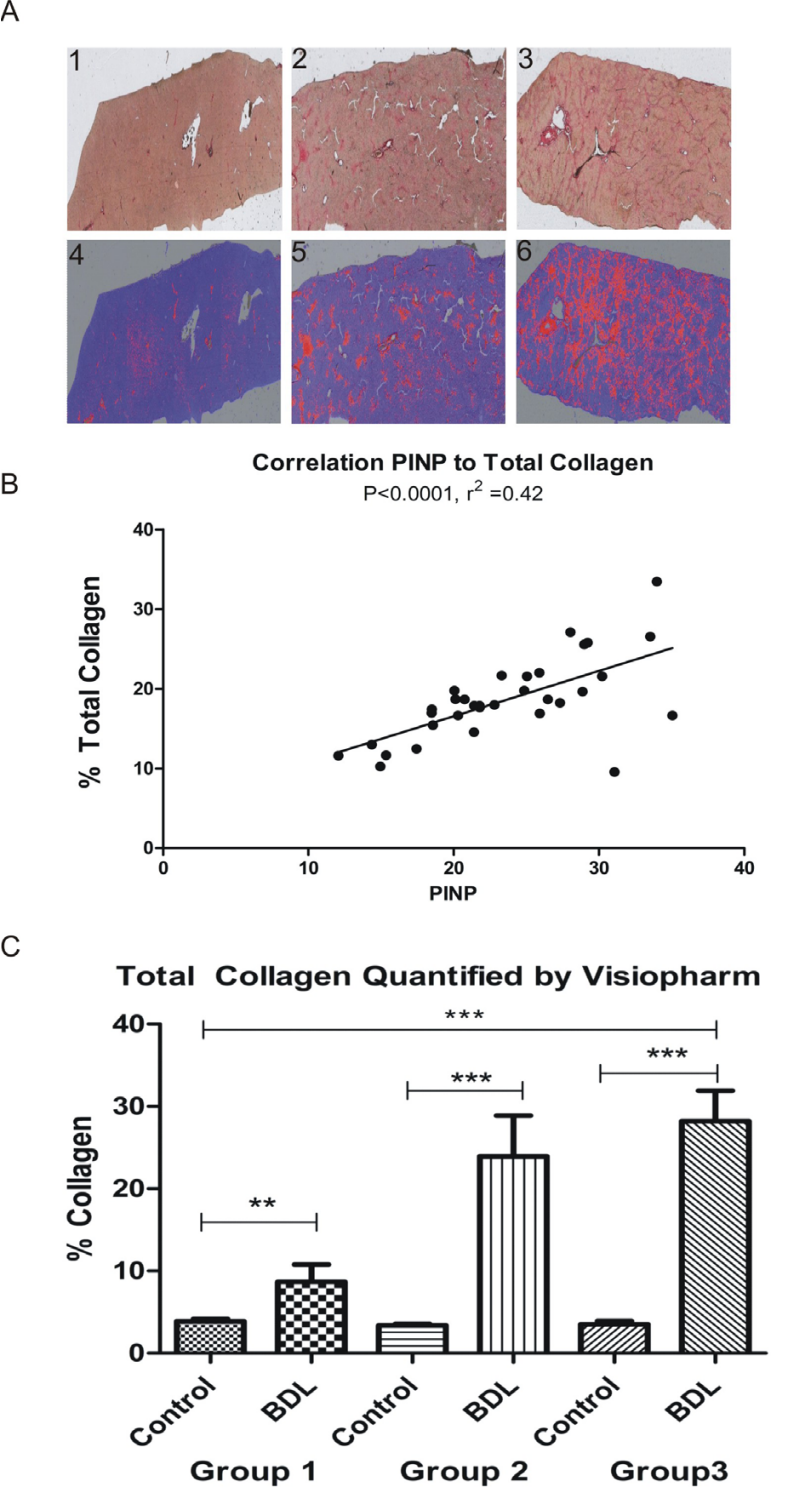
**Histology and collagen**. **(a) **Top panel: histology sections from bile duct ligated (BDL) or sham-operated rats stained with Sirus Red as viewed by conventional light microscopy (top row) and after masking (bottom row), showing red colour in areas with collagen and blue in the parenchyma. Bottom panel: Masked histology sections for quantifying total collagen content (red colour) in the liver. **(b) **Correlation of serum procollagen type I N-terminal propeptide (PINP) and percentage liver content collagen. **(c) **Total collagen quantified by Visiopharm software for each group.

The extent of liver fibrosis was evaluated quantitatively by measuring the extent of Sirus Red staining, as an indication of the total collagen deposition. Sections stained with Sirus Red and quantified using Visiopharm software revealed increased collagen content over time in the BDL-operated rats (Figure 3a). The red colour in the mask representing collagen was quantified using the same software (Figure 3b) and confirmed a significant increase in total collagen content in BDL-operated rats compared with sham-operated rats (group 1, *P *= 0.008; group 2, *P *= 0.0006; group 3, *P *< 0.0001).

### Correlations of measured quantitative parameters

To further examine the relationship between PINP and total collagen increase in liver fibrosis the two parameters were evaluated by quantitative histology and linear correlation was performed. This revealed a highly significant correlation with R^2 ^= 0.42 and *P *< 0.0001 (Figure 3c). This suggests that collagen type I is an important molecule in the pathogenesis of liver fibrosis. No correlation between changes in PINP levels and osteocalcin levels was detected in the two treatment groups (data not shown).

## Discussion

Matrix remodelling is an integrated process of tissue development, maintenance and repair. The key constituents of ECM in the liver are collagens and proteoglycans, each with their own unique biophysical properties. In fibrosis, the most abundant molecules in the ECM are various forms of collagens, in particular type I and III, as well as a range of proteoglycans. During fibrogenesis, type I collagen increases significantly and turnover markers of this protein may be potential biomarkers of liver tissue metabolism.

The present study is the first to demonstrate that hepatic collagen formation in BDL rats can be measured in the circulation. Rises in PINP serum levels of up to 52% in BDL rats, indicating increases in type I collagen formation, were shown to be correlated with the extent of liver fibrosis (R^2 ^= 0.42, *P *< 0.001). Histological staining showed extensive liver fibrosis in the BDL groups, with a total collagen content of more than 20% in some rats, and an average increase over control of more than 10 times. Immunohistochemistry of type I collagen confirmed extensive deposition of type I collagen in disease-affected, but not healthy, livers. Type I collagen was mainly present around neo-bile ducts. Previous investigators have demonstrated that type I collagen is increased significantly in various animal models of liver fibrosis [4,5,8,11], and the current data are in agreement with the notion that type I collagen is the predominant collagen in liver fibrosis.

Both bone and fibrotic livers are major sources of type I collagen turnover and it is important to distinguish between sources when trying to determine the relative contribution of each tissue to systemic levels of circulating PINP. The organic matrix in bone consists of approximately 90% type I collagen, while the remaining 10% is composed of proteoglycans and numerous non-collagenous proteins, of which osteocalcin and osteonectin constitute 40% to 50% [22,23].

Bone is a high turnover tissue, resulting in the entire skeleton being totally remodelled in approximately 10 years. Different experiments have suggested that PINP is a valid bone-formation marker [12], however the present and other studies suggest that PINP is also present in many other tissues such as skin, lung, tendons and liver [18,24,25]. Our experiment suggests that PINP was specific for the hepatic injury, since the bone-specific marker osteocalcin did not increase in BDL rats or in the sham-operated rats. In addition, no correlation between changes in PINP levels and osteocalcin levels was detected in the two treatment groups (data not shown), indicating that the increased PINP associated with liver fibrosis was not correlated to bone formation measured by osteocalcin.

PINP is currently used to monitor the response to parathyroid hormone (PTH) treatment for osteoporosis [12]. Our findings may have two implications for such monitoring: (1) PINP levels may not be bone specific and as such may overestimate efficacy of PTH therapy, (2) PTH may have extraskeletal effects on many tissues in which collagen type I is turned over. On the basis of our findings, further investigation is warranted to determine whether more bone-specific markers, such as osteocalcin, may more accurately reflect bone metabolism in response to PTH treatment.

Bone loss has been reported previously to be a complication of liver fibrosis [26-29]. Total bone mass results from a balance between bone resorption and bone formation. In the present study we did not detect from osteocalcin measurements large changes in bone formation, even in the presence of severe liver fibrosis. This suggests that the decreased bone mass and quality observed by other investigators [27,30] in liver fibrosis may be driven by an increase in bone resorption, as previously suggested in patients with liver cirrhosis secondary to viral infection [31]. This is in agreement with the notion of poor nutrient uptake in cases of liver fibrosis, and subsequent increased compensatory calcium reabsorption from the bones, leading to increased bone resorption [30]. Interestingly, the decrease in bone formation in the BDL-operated animals in group I, which may be considered the more acute response to surgical treatment, showed a decrease in bone formation, whereas the other groups did not display differences. This is somewhat in agreement with earlier findings demonstrating decreases in bone formation in patients [32] and in animals [30].

The present experiments were conducted in mature rats, which is the gold standard for bone metabolic diseases due to the very high bone turnover in younger rats [12]. This unique experimental design in the rat liver fibrosis model allowed for better discrimination of the tissue source of the biochemical marker than may have been possible with younger animals. It has previously been published that the collagen turnover profile in rats is highly dependent on age, in which, for example, collagen type II degradation marker levels decreased from 850 μg/mmol at 1 month of age to 1 μg/mmol at 6 months of age [33]. Similar age-dependent profiles have been reported for type I collagen formation [34].

Our study has some limitations. As PINP propeptides in part are cleared by liver endothelial cells, and these cells may be injured secondary to inflammation due to the BDL, a part of the observed increase could be due to failure of damaged endothelial cells to clear PINP [35]. As an example, patients presenting with alcoholic liver fibrosis with acute inflammation, N-terminal propeptide of type III collagen (PIIINP), a marker of type III collagen formation, is significantly increased, whereas in cirrhosis, PIIINP is only moderately increased [8]. This increased level of PIIINP may both be due to impaired clearance but also the inflammation process itself, as collagen type III is present in arteries and synovium that are highly inflamed. As the level of any serological biochemical marker reflects a balance between formation and clearance, this issue deserves further attention.

## Conclusions

In summary, we provide evidence that a collagen type I turnover product, PINP, is a potential marker for monitoring fibrosis during chronic liver disease. Additional research in well controlled clinical settings is needed to further investigate this finding.

## Competing interests

SSV, EV, A-CB-J and MAK are employees of Nordic Bioscience. MAK owns stocks and shares in Nordic Bioscience.

## Authors' contributions

SSV designed the study, measured PINP and performed quantitative histology. EV performed quantitative histology. A-CB-J performed immunohistochemistry and participated in design of the study and analysis of data. GT and BV drafted the first versions of the manuscript and discussed data analysis. MAK and SSV participated in all aspects of study design and manuscript drafting. All authors approved the final version of the manuscript.
